# Hematopoietic Stem Cell Transplantation in Neuromyelitis Optica-Spectrum Disorders (NMO-SD): State-of-the-Art and Future Perspectives

**DOI:** 10.3390/ijms21155304

**Published:** 2020-07-26

**Authors:** Giulia Ceglie, Laura Papetti, Massimiliano Valeriani, Pietro Merli

**Affiliations:** 1 Department of Hematology/Oncology, Cell and Gene Therapy, Bambino Gesù Children’s Hospital, 00165 Rome, Italy; giulia.ceglie@opbg.net; 2 Department of Neurology, Bambino Gesù Children’s Hospital, 00165 Rome, Italy; laura.papetti@opbg.net (L.P.); Massimiliano.valeriani@opbg.net (M.V.)

**Keywords:** NMO, NMOSD, HSCT, monoclonal antibodies, multiple sclerosis

## Abstract

Neuromyelitis optica (NMO) and neuromyelitis optica spectrum disorders (NMOSD) are a group of autoimmune inflammatory disorders of the central nervous system (CNS). Understanding of the molecular basis of these diseases in the last decades has led to an important improvement in the treatment of this disease, in particular, to the use of immunotherapeutic approaches, such as monoclonal antibodies and Hematopoietic Stem Cell Transplantation (HSCT). The aim of this review is to summarize the pathogenesis, biological basis and new treatment options of these disorders, with a particular focus on HSCT applications. Different HSCT strategies are being explored in NMOSD, both autologous and allogeneic HSCT, with the new emergence of therapeutic effects such as an induction of tolerance to auto-antigens and graft versus autoimmunity effects that can be exploited to hopefully treat a disease that still has prognosis.

## 1. Introduction

Neuromyelitis optica (NMO) and neuromyelitis optica spectrum disorders (NMOSD) are a group of immune-mediated diseases characterized by inflammatory damage of the myelin and axonal damage. Typically, the most frequently affected areas are the optic nerve and the spinal cord. From a serological point of view, the most specific marker of the disease is the presence of IgG antibodies against aquaporin-4 (AQP4) [1].

The NMO is estimated to represent approximately 1% of CNS inflammatory demyelinating diseases in Europe, with an estimated prevalence of 1–2/100,000 [1]. NMO is more frequent in women than in men, and it usually begins in young adults even if an onset in childhood is possible [2,3].

Some familiar forms of NMO have been described, but they cannot clearly be linked to any disease-specific genetic mutation and AQP4 polymorphism [4].

The diagnosis of NMO/NMOSD requires a history of typical clinical presentations and findings on neuroimaging. The analysis of cerebrospinal fluid (CSF) and the research of AQP4-IgG serum autoantibody are mandatory [5].

NMO therapy is often a challenge for the clinician, and it is based on relapse therapy (corticosteroids and plasmapheresis) and preventive treatment. To date, there is little evidence on which type of treatment is most effective for controlling NMO. The most commonly used drugs are immunosuppressors such as azathioprine, rituximab, methotrexate or mycophenolate [6].

However, in recent years, new scenarios have opened up with new monoclonal antibodies for NMOSD therapy [7]. Scientific evidence from case reports and series also support the use of hematopoietic stem cell transplantation for forms refractory to standard treatments [8,9,10]. These data will be reviewed in the present manuscript.

## 2. Materials and Methods

The authors conducted a literature search describing the issue of Hematopoietic Stem Cell Transplantation (HSCT) in NMOSD. Research studies were selected based on research topics. The search terms used in PubMed were “HSCT” or “bone marrow transplantation” and “NMO” or “NMOSD”, “Allogeneic HSCT”, “Autologous HSCT” and “haploidentical HSCT”. Only papers written in English were considered, and those published from the year 2000 up to June 2020 were mostly selected. We included reviews, case series and research studies that were classified according to their relevance. No abstracts were included.

The information found in the selected studies, with particular attention to epidemiology, histological and biological characteristics, symptoms, diagnosis and treatment were carefully evaluated and are described and discussed in the following sections.

## 3. Pathogenesis of NMOSD

Brain and spinal lesions of NMO patients show the presence of necrotic lesions with infiltration of neutrophils and eosinophils, deposits of IgM and IgG, and complement activated fractions on the wall of blood vessels as well as reduced expression of AQ4 on astrocytes [6]. These data suggest the involvement of different mechanisms in the pathogenesis of NMOSD. The damage caused by these immune-mediated mechanisms involves both grey and white matter, including axons and oligodendrocytes. Demyelination and loss of neuronal cells occur as a consequence of astrocytic damage [6,7].

The immune target is represented by aquaporin-4 (AQP4). The water channel AQP4 is the target of the immunoglobulin G autoantibody (AQP4-IgG) in NMOSD. The AQP4-IgG is an IgG1 autoantibody, and it has been identified in the serum of about 75% of NMOSD patients. Detection of AQP4-IgG is less sensitive in cerebrospinal fluid (CSF) than in serum, suggesting that a large proportion of AQP4-IgG originates in peripheral lymphoid tissues. Therefore, the most effective test for seropositive NMOs is the determination of serum Ig AQ4 [11]. The AQP4-IgG titre also correlates with disease activity and relapses [12]. The presence of AQ4 IgG in serum is highly predictive of evolution in NMO in patients with optic neuritis or transverse myelitis and is associated with a high risk of future relapse. Clinical relapses are generally preceded by a progressive increase in the Ig AQ4 titre, and the latter levels decrease during immunosuppressive treatments [12].

Another type of antibody called MOG-Ab (myelin oligodendrocyte glycoprotein antibody) has been identified in approximately 5–10% of NMOSDs negative for AQP4-Ab [13]. The MOG-IgG is considered a potentially pathogenic biomarker for a similar but separate disease entity (anti-MOG syndrome) rather than for a subgroup of NMOSD patients [14].

AQP4 constitutes the main mechanism for the passage of transmembrane water at the brain, spinal cord, retina, inner ear and musculature. It is the most abundant aquaporin in the brain of mammals, and it is expressed at major concentrations near the blood–brain barrier and at the level of the pedicels of astrocytes [15]. It is also present at the level of ependymal cells and, at lower level concentrations, at the level of endothelial cells of the brain tissue and of the nuclei supraoptics of the hypothalamus and periventricular structures [16]. On the pedicels of astrocytes, AQP4 is associated with the potassium channel (Kir 4.1), which is involved in the regulation of extracellular K concentration [17]. In addition, its localization is also associated with the molecule involved in the transport of glutamate (GLT-1). Glutamate represents one of the main excitatory neurotransmitters of astrocytes [17].

Such strategic localization, in association with Kir 4.1 and GLT-1 at perivascular levels and subependymal, gives AQP4 a potential role of severe damage at the level of myelin and axons of vulnerable brain areas, such as the optic nerve or the spinal cord [17]. Also significant is the fact that the distribution of areas rich in AQP4 in the CNS (the central part of the spinal cord, the hypothalamus, the periventricular areas and periaqueductal areas) has a high correspondence with the location of the lesions in the NMO [16].

AQP4-IgG crosses the blood–brain barrier (BBB) and binds to the AQP4 proteins expressed by astrocytes. This process activates the complement and other effector cells which are then responsible for the cytotoxicity that damages the astrocytes [18]. Complement activation and astrocyte damage recruit inflammatory cells such as eosinophils, neutrophils and macrophages that locally determine the release of cytokines (IL-17 and IL-8), proteases and radicals that contribute to both vascular and parenchymal damage [18,19]. In particular, the damage of the BBB promotes further entry of AQP4 into the CNS [19]. However, the presence of plasma AQP4-IgG alone is insufficient to damage the BBB. This is also suggested by the evidence that the severity of NMO is not correlated with serum AQP4-Ab levels [20] and that the injection of IgG of patients with NMO into the brain of naïve mice does not cause NMO-like lesions [21]. These data therefore suggest the involvement of other immunological mechanisms (e.g., cellular immunity) in combination with AQP4-IgG in the pathogenesis of the disease. In particular, B cells seems to play a key role in the NMO pathology and, on this basis, many therapies for NMO deplete B cells or modulate their activity. Strategies such as plasmapheresis, depletion of B lymphocytes by rituximab and interference with IL-6 (which acts also as a growth and differentiation factor of B lymphocytes) are the most effective strategies to prevent relapses [22].

AQP4-Abs are produced by B lymphocytes with specific CD19int, CD27high and CD38high phenotypes which are selectively increased in the blood of subjects with NMO, especially during relapse [23]. B cells are involved in the pathogenesis of NMO through various mechanisms [20]. Both central and peripheral tolerance defects should be responsible for the presence of autoreactive B lymphocytes. Moreover, B cells induce the activation of T cell responses trough mechanisms of antigen presentation or cytokine secretion [22,24]. In a second phase of NMO pathogenesis, cells of the adaptive immune system extravasate into the affected tissue. Myeloid antigen (Ag)-presenting cells (APCs) process AQP4 and present Ag to CD4-T cells [22,24]. Consequently, the activated AQP4-specific T cells damage the BBB and allow further entry of AQP4-IgG and other effectors into tissues containing astrocytes expressing AQP4 [23]. In NMOSD, activated T helper lymphocytes present a Th17 phenotype dominance [25]. This differentiation is promoted by the high production of IL-6 that is observed in patients with NMOSD [23]. In turn, the Th17 activation causes the production of cytokines such as IL-17, Tumor Necrosis Factor-alpha (TNF) and granulocyte-macrophage colony-stimulating factor (GM-CSF) which promote tissue damage [26,27,28]. Moreover, activated T cells cross-activate antigen-specific B cells that mature into plasma cells and memory B cell producing AQP4-Abs. The antibodies in turn keep the initial phase of this immune and inflammatory cascade active by amplifying the astrocytic damage [28]. The binding between AQP4-IgG and AQP4 on astrocytes causes complement activation through the classical pathway [15]. Complement-dependent cytotoxicity combined with antibody-dependent cell-mediated cytotoxicity (ADCC) and internalization of the glutamate transporter EAAT-2 cause astrocytes to lose the ability of osmotic regulation and glutamate uptake [29]. Finally, astrocytic damage results in a lack of support for oligodendrocytes and neurons with demyelination and axonal loss responsible for a patient’s disability [15,28].

## 4. Clinical Features and Diagnostic Criteria

NMO has long been considered a disease characterized clinically by the association of recurrent episodes of optic neuritis and longitudinally extensive transverse myelitis (LETM). The discovery of the AQP4-IgG and the description of new clinical and neuroradiological pictures allowed to expand the phenotype with the definition of NMOSDs. The spectrum of NMO therefore refers to clinical pictures in which the positivity of AQP4-IgG is associated with other clinical pictures besides optic neuritis and myelitis such as diencephalic, brainstem or other cerebral syndromes [1,5]. In 2015, an international consensus was published which established the criteria for the diagnosis of NMOSD. The six core clinical pictures included 1) optic neuritis; 2) acute myelitis; 3) area postrema syndrome, which manifests with persistent hiccups or nausea and vomiting; 4) acute brainstem syndrome; 5) symptomatic narcolepsy or acute diencephalic clinical syndrome; and 6) symptomatic cerebral syndrome. The last two clinical manifestations required the simultaneous presence of NMOSD-typical brain lesions detected in MRIs [5]. The episodes of neuritis are more commonly unilateral than bilateral, but the presence of bilateral simultaneous optic neuritis is high specific for NMO [2]. In patients with positive AQP4-IgG, a clinical core is sufficient for diagnosis. In patients without evidence of serum AQP4-IgG, the diagnosis can be established only if there are two clinical events with core features [5].

**Figure 1:**
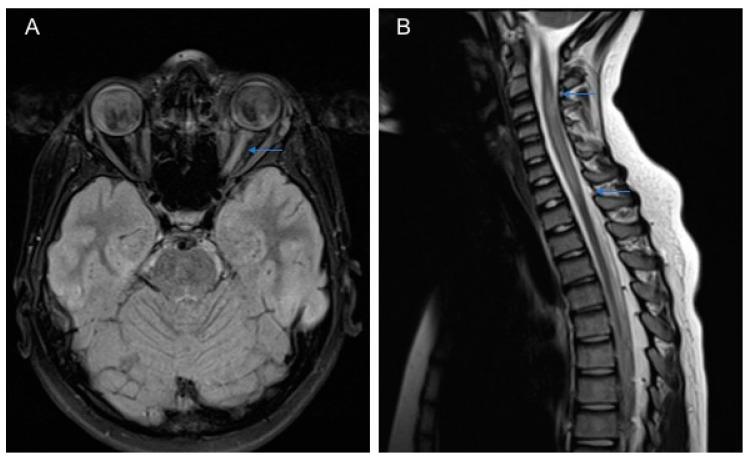
MRI of a 11-year-old patient with Neuromyelitis optica (NMO): (**A**) the brain axial Flair-T2 weighted image shows hyperintensity of the left optic nerve and (**B**) the spinal T2 weighted image shows cervical hyperintense lesions extending longitudinally from C3 to C7 (longitudinally extensive transverse myelitis (LETM).

Magnetic resonance imaging (MRI) of the brain is often diriment for the diagnosis of NMOSD. The most suggestive MRI pictures include an alteration of the optic nerve (Figure 1A) or a spinal cord injury that extends for more than three metamers (LETM) (Figure 1B). However, in other cases, lesions in other locations such as medulla/area postrema lesions or periependymal brainstem can be found [30].

The course of NMO should be monophasic or relapsing [2]. The monophasic course occurs in about 25% of patients, with concomitant involvement of either unilateral/bilateral optic neuritis and a single episode of transverse myelitis [31]. About 75% of patients develop a relapsing course with recurrent optic neuritis and myelitis with significant increasing disability [31]. The most frequent disabilities included permanent vision loss and motor dysfunctions. Subjects who are younger at disease onset have a higher probability of visual disability; otherwise, patients who are older at onset have a higher chance of motor disability [13].

In patients with anti-MOG antibodies, the most frequent clinical presentations include acute disseminated acute encephalomyelitis (ADEM) with optic neuritis (ADEM-NO) or relapses of optic neuritis, although in most cases, the course remains monophasic [14].

## 5. Therapy

NMOSD therapy is based on the acute treatment of relapses and therapy that prevents relapses.

The acute treatment of relapses is based on the use of high dose corticosteroids (methylprednisolone), intravenous immunoglobulins and plasmapheresis [32].

Therapy that prevents relapses and modifies the course of the disease can use different drugs. Generally, the most used immunosuppressant drugs are azathioprine (AZA) and mycophenolate (MMF) [11,33,34]. Despite the effectiveness of these drugs, their long-term use can be associated with numerous adverse effects [35]. Their use has been gradually overshadowed with growing evidence of the efficacy of rituximab (RTX) for the treatment of NMOSD [35,36,37,38,39].

Positive MOG patient relapses are treated in the same way as NMOSD relapses. For chronic treatment, however, therapeutic strategies include long-term treatment with low doses of prednisolone; monthly intravenous immunoglobulins cycles; as well as, for refractory patients, immunosuppressive therapies with rituximab, mycophenolate mofetil, methotrexate or azathioprine [13,14]. Notably, for patients with NMOSD, there is a general consensus that treatment was effective regardless of serostatus (AQP4- IgG positive or negative) [11,33].

RTX is a monoclonal antibody directed to CD20 antigen on B cells, and it causes a rapid depletion of circulating CD20^+^ B cells [36]. RTX has been often used now as first-line treatment in highly active NMOSD [40,41]. In patients affected by NMOSD, RTX causes a removal of B cells as antigen-presenting cells and it also produces a reduction in the CD20^+^ early plasmablast population generating AQP4-IgG [42].

RTX has been progressively used as a first-line therapy with a suggested better control of disease activity compared to AZA and possibly MMF [35,37,38,43]. Finally, the efficacy and safety of RTX in NMOSD has recently been evaluated in a multicentre, randomized, double-blind, placebo-controlled (RIN-1) study. This study included 38 patients AQP4 antibody-positive treated with RTX (375 mg/m²). RTX prevented relapses for 72 weeks in patients with NMOSD AQP4 positive [44]. RTX has acceptable tolerance, reduces the relapse frequency and improves disability in most patients with NMOSD [45]. Maintaining the depletion of memory B cells through repeated treatment courses may be pivotal to the clinical effects of RTX in patients with NMO [45].

Recently, the therapeutic scenario has opened up to three new monoclonal antibodies subjected to analysis in clinical trials: eculizumab [46], inebilizumab [47] and satralizumab [48].

In 2019, The U.S. Food and Drug Administration (FDA) and the European Medicines Agency (EMA) approved eculizumab injection for intravenous use for the treatment of NMOSD in adult patients who were AQP4 antibody positive. Eculizumab is a long-acting humanized monoclonal antibody targeted against complement C5. It inhibits the cleavage of C5 into C5a and C5b and hence inhibits deployment of the terminal complement system [49]. The rationale behind testing a complement inhibitor in NMOSD is based on the pathology of NMO lesions showing extensive complement deposition, and it derives from the study of the role of complement in pathogenesis of the disease [50]. The effectiveness of eculizumab for the treatment of NMOSD was demonstrated in the phase III PREVENT study (prevention of relapses in neuromyelitis optica). This randomized, double-blind, placebo-controlled study included 143 patients with anti-AQP4-positive NMOSD. Eculizumab was administered intravenously at doses of 900 mg weekly for the first four doses and then 1200 mg every 2 weeks from the following week. At 48 weeks, 98% of eculizumab-treated patients had no relapse compared to 63% of placebo-treated patients. This effect was observed for 144 weeks of treatment, with 96% of patients receiving eculizumab without relapses compared to 45% of patients in the placebo arm. Eculizumab also reduced hospitalization rates and the need for treatment of acute attacks with corticosteroids and plasma exchange [46]. Notably, eculizumab is associated with an increased susceptibility to opportunistic meningococcal infection (*N. meningitidis*) (EMA 2019).

Inebilizumab is a humanized, affinity-optimized, afucosylated IgG1 kappa monoclonal antibody that acts as an anti-CD19 monoclonal antibody that targets circulating B cells, differing from anti-CD20 therapy by depleting pro-B cells and plasmablasts in addition to pre-B cells through memory B cells [51].

Inebilizumab has recently shown efficacy in a randomized double-blinded, placebo-controlled clinical trial with 174 participants receiving inebilizumab and 56 participants receiving a placebo. Intravenous inebilizumab or placebo was administered on days 1 and 15 (the total dose of inebilizumab in the randomised controlled period was 600 mg, with no further doses occurring after day 15 in this study period). The trial tested inebilizumab as a monotherapy in NMOSD patients with or without AQP4-IgG, and it showed that 12% of participants receiving inebilizumab had an attack versus 39% participants receiving the placebo (*p* < 0.0001). The secondary outcome of decreasing disability worsening compared to placebo was also reached. In detail, AQP4-IgG positive patients were those with the greatest evidence of efficacy. The most frequent side effects were infusion reactions [47]. To date, inebilizumab is under FDA review for approval use in NMOSD [52].

Satralizumab is a monoclonal antibody that acts by antagonizing IL-6, and it was evaluated in a randomized, double-blind, placebo-controlled trail (phase 3) enrolling 83 patients with NMOSD (41 in the satralizumab group vs. 42 in the placebo group). Satralizumab was administered subcutaneously at baseline and then later at 2 weeks and at every 4 weeks. The concomitant use of stable immunosuppressant treatment was allowed. The results of the trial showed that relapse occurred in 20% of patients under satralizumab treatment vs. 43% of the patients with placebo (hazard ratio, 0.38). At 48 weeks, 89% on satralizumab and 66% on placebo were relapse-free. In the analysis of subgroups, the AQP4-positive subjects presented a relapse in 11% of satralizumab patients vs. 43% of placebo patients. Adverse events were more frequently observed in the placebo group [48]. Another drug acting against IL-6 is a humanized anti-IL-6 receptor called tocilizumab, which inhibits both classical and trans IL-6 signalling. To date, tocilizumab showed efficacy only in a pilot study conducted on few cases of NMO and it is considered as a third-line treatment for severe cases [53,54].

Even though these new immunotherapeutic strategies hold steady progress in the treatment of NMOSD, a curative approach is not yet available and the vast majority of patients have a temporary control of the disease and require multiple lines of therapy. Hematopoietic Stem Cell Transplantation (HSCT) fits into this context, providing an alternative and possibly curative form of immunotherapy.

## 6. Historical and Biological Basis of HSCT in Neurological Autoimmune Disorders

Hematopoietic Stem Cell Transplantation (HSCT) is a complex procedure that includes the substitution of the host hematopoietic system through chemo- and/or radiotherapy with a new one, thus completely erasing the immune system. The first and most used applications of the procedure were in the treatment of onco-haematological disease [55], but its role in the cure of autoimmune diseases (AD) has recently emerged and has been demonstrated in several diseases, including neurological ones [56]. According to the donor of the hematopoietic stem cells (HSCs), HSCT procedures can be divided into autologous (auHSCT) or allogeneic (alHSCT). In auHSCT, the cells of the patients are harvested and frozen, and after a radio- and/or chemotherapy regimen, the cells are reinfused in the patients (that acts both as the donor and the recipient) with the aim of rescuing him/her from long-lasting cytopenia. AlHSCT, on the other hand, involves two subjects: the (healthy) donor and the (patient) recipient. So far, in the treatment of severe autoimmune diseases, auHSCT has been preferred over alHSCT mainly because of the lower toxicity due to the absence alloreactivity (i.e., of Graft-versus-Host Disease (GVHD) [57] and lower Transplant Related Mortality (TRM), defined as death due to causes unrelated to the underlying disease but directly consequent to the transplant procedure [58]).

The biological bases of HSCT treatment is that the neuroinflammation is due to an immune system error dependent on immunological memory. HSCT aims to destroy the immune system, thus erasing its erroneous response towards self-antigens and allowing the reconstitution of a new, self-tolerant immune system [59]. In this context, HSCT stands as a potentially curative treatment with the notable consequence that the patients potentially would not require additional therapy after the procedure.

In the early 1990s, the first studies on the effects of both autologous and allogeneic HSCT for experimental autoimmune encephalomyelitis (EAE) were conducted in rodent models [60,61]. In these studies, both immune-ablation through Total Body Irradiation (TBI) and cyclophosphamide (Cy) followed by infusion of syngeneic bone marrow from healthy rats or different resistant strains were tested and proved to be efficient in both clinical and histopathological terms [62,63]. Indeed, the treated animals did not reach the state of paralysis like non-transplanted animals; moreover, the histopathological specimen obtained showed no significant inflammation. Concerning the different role of different type of HSCT, it was observed that auHSCT was only effective when performed very early after induction of the disease, thus proving to be effective only in the first stages of the disease [64]. Taking the first steps from these animal studies in 1995, the first auHSCT was performed in a patient with Multiple Sclerosis (MS) [65], shortly followed by numerous other experiences [66,67,68]. These seminal efforts, although at first sometimes disappointing, over the course of less than two decades led to the first consensus recommendation in 2012 that HSCT should be considered as a therapeutic option at second line or beyond for patients with relapsing-remitting MS who deteriorate despite standard therapy [69]. Although most of the experiences on HSCT in autoimmune neurological diseases was achieved in MS for prevalence reasons, in the following years, the field extended to other diseases, such as NMO and NMOSD.

## 7. Autologous HSCT in NMOSD

AuHSCT does not carry the same TRM risk as alHSCT, and thus, it is a relatively safe procedure in specialized centres. The first step of auHSCT is mobilization: to mobilize HSCs from the bone marrow into the peripheral blood, different drugs (most frequently, granulocyte colony-stimulating factor (GCSF)) are administered. The mobilized stem cells are then harvested through leukapheresis, and the high-dose conditioning can be started: chemotherapy (and less frequently radiation) are given to delete the self-reacting immune system of the patient. Since lymphocytes (of both T and B lineage) are the population responsible for the abnormal immune reaction, conditioning regimens used frequently to incorporate antibodies (e.g., antithymocyte globulin (ATG) and/or rituximab) able to eliminate these cells both in the recipient. Finally, autologous HSCs are reinfused; since ATG and rituximab half-lives are prolonged, their persistence in the patient delete in vivo T and B lymphocytes present in the graft. It is important to notice that the therapeutic potential of the procedure is not dependent on the HSC product. The actual therapeutic phase is the conditioning that acts as a highly active immunosuppressive therapy that allows for the complete reset of the immune system. HSCs are, in this case, a mere support product that speeds the hematopoietic recovery that may not be achieved or delayed in the absence of a reinfusion.

The first report of auHSCT in NMO dates back to 2010 when Peng and colleagues performed it in a 23-year-old female patient with a very severe disease course characterized by paraparesis, vision loss, radicular pain and dysesthesia [70]. Stem cells were harvested from peripheral blood, and the procedure was performed 2 years after diagnosis. After 12 months, she was stable without any further relapses. Motor and sensory functions were restored except for visual acuity (but atrophy of the optic nerve was present before treatment). Also, imaging abnormalities appeared to be improved.

In 2014, the European Group for Blood and Marrow Transplantation Autoimmune Diseases Working Party (EBMT ADWP) presented data from a retrospective multicentre study [9] with 16 patients diagnosed with NMO or NMOSD mainly treated with the BEAM (BCNU, Etoposide, Ara-C, Melphalan)-ATG regimen followed by HSC infusion. Three cases remained progression- and treatment-free, while in 13 patients, anti-AQP-4Ab antibodies persisted, leading to relapse requiring further treatment. Moreover, one patient died from disease progression 14 months after HSCT.

Recently, Aouad and colleagues reported on a 47-year-old female patient that underwent autologous HSCT with a disease duration of 11 years [71]. Rituximab was administered as part of the conditioning regimen (cyclophosphamide + Anti-thymocyte Globulin, ATG). At 12 months follow-up, she had a sustained clinical, radiological and immunopathological remission. Also, recent data from the Northwestern University [72] supported the use of a Cy-based (ATG and rituximab) auHSCT to obtain a prolonged drug-free remission in 12 patients with NMOSD; all of them achieved clearance of anti-AQP-4Ab. Of the 12 patients, 11 were female, 8 were Caucasian and 4 were African American. Mean age was 42 years, and mean duration of disease before HSCT was 84 months. Before transplant, 11 had clinical attacks of optic neuritis, 12 had myelitis, 1 had area postrema syndrome and 11 of 12 were AQP4-IgG-positive. The mean pretransplant EDSS (Expanded Disability Status Scale) score was 4.3. The patents were followed up for 5 years and achieved neurological improvements; in particular, EDSS score improved from a baseline mean of 4.4 to 3.3, and quality of life significantly improved. The median day of white blood cell engraftment (defined as neutrophil count >1000/μL) was day +9. The treatment appeared to be very well tolerated, with few toxicities and with only 1 inpatient infection (*Clostridium difficile*). Grade 3 toxicities were reported, the most frequent one being hypophosphatemia, followed by neutropenic fever, hypocalcemia, nausea and vomiting. No grade 4 toxicities were reported. The number of infections after HSCT was 0.18 per year per patient. Interestingly, 2 patients developed new autoimmune diseases: one developed myasthenia gravis that occurred with NMOSD relapse, and the other one developed hyperthyroidism.

Other reports have investigated the outcome of auHSCT in the context of NMOSD, with less structured conclusions [73,74]. A Chinese study [73] treated 20 patients with Opticospinal Multiple Sclerosis (OSMS), a disease comparable to NMOSD, with auHSCT. Modified BEAM conditioning regimens were administered (tiniposide, melphalan, carmustin and cytosine arabinoside). Outcome was evaluated with ExpandedDisability Status Scale (EDSS) scores; for all patients, the overall EDSS score decreased significantly after transplantation while visual functions had no significant improvement. Confronting the OSMS relapse rate with conventional multiple sclerosis, progressive OSMS had a higher relapse rate. Matiello and colleagues [74] described a case of a woman with relapsing NMO and who experienced a relapse of myelitis 4 months after auHSCT for a lymphoma that developed while receiving AZA therapy. Also, a marked increase of NMO-IgG was documented coinciding with the relapse.

All this considered, the recently published EBMT-updated guidelines on the indication of auHSCT in MS and other immune-mediated neurological disorders, including NMO and NMOSD, recommend auHSCT in patients with refractory NMO (level II recommendation) [10]. What emerges from the available data is that auHSCT can reduce inflammation in NMO especially in the long term but that a number of patients will relapse within 5 years. Conditioning regimens containing rituximab might improve prognosis but the very few cases described prohibit drawing firm conclusions.

## 8. Allogeneic HSCT in NMOSD

AlHSCT is less explored in AD and mainly restricted to the paediatric setting, this restriction mainly deriving from the lower risk of TRM and, in general, of transplant-related morbidities in children compared to adults [75]. Also, the more frequent indication for alHSCT is immune cytopenia, mainly affecting paediatric patients [76].

AuHSCT is a relatively safe and relatively effective treatment for NMOSD. In particular, auHSCT has been proven to maintain a good temporary control of the disease but scarce control in the long term. These issues are of utmost importance when considering treatment options in very young or paediatric patients, when a stable and durable control of the disease is fundamental in light of the relatively long life-expectation compared to adult patients.

From a biological point of view, the main difference between alHSCT and auHSCT is the fact that a healthy donor is required as a source of HSCs. HSCs can be collected from the peripheral blood after a mobilization phase or can be harvested from the bone marrow of the donor. The patient undergoes a conditioning regimen, usually more intensive than those used in auHSCT in order to cross the HLA (Human Leukocyte Antigen) barrier, and it is followed by infusion of the HSCs of the donor. In this setting, the conditioning regimen not only has the role of resetting the immune system of the patient but also is necessary to “make space” for the new hematopoietic system to avoid the risk of rejection and to allow for the engraftment of the new cells. Indeed, differently from auHSCT, the bidirectional alloreactivitiy (namely host-versus-graft and graft-versus-host), which depends on several factors (including HLA disparity, age, sex mismatch, intensity of the conditioning, etc.), can cause graft rejection or graft-versus-host-disease (GVHD). This is why, in alHSCT, prophylaxis of GVHD is necessary, consisting either of drugs (mostly methotrexate and calcineurin inhibitors) and/or manipulation of the graft.

As for the donor types, an HLA-matched family donor (MFD) is generally considered the best option, because of prompt availability and lower risk of GVHD, but such a donor is available in less than 25% of cases [76]. Also, fully matched unrelated donors (MUD) have been used in the setting of AID (Auto-Immune Diseases), while alHSCT from haploidentical donors (i.e., those sharing only one haplotype, thus, with a high degree of HLA mismatch) is still considered highly experimental. Potential HLA-haploidentical donors are biological parents, biological children, full or half siblings, or even other family members. One of the main advantages of this type of HSCT is (i) the fact that virtually every patient has a haploidentical donor and (ii) its fast availability (since the donor is usually close to the patient and highly motivated). The main disadvantage is HLA mismatch, thus increasing the risk of both graft rejection and severe GVHD. Because of these issues, innovative prophylactic measures have been used, including graft manipulation in order to deplete the alloreactive T cells (T-cell depleted transplants) [77] or the use of posttransplantation cyclophosphamide (T-cell repleted transplants) [78].

Limited experiences are available regarding the use of alHSCT in NMOSD, but they appear to be promising. The first report of the use of alHSCT in patients with NMO is that of Greco et al., who successfully treated with al-HSCT the first 2 patients (see also Table 1) [79]. Both patients had already undergone auHSCT with insufficient results. One of the 2 patients received an HLA identical HSCT from an MFD, while the second patient received the transplant from an unrelated donor with a 9/10 HLA match. In both cases, the conditioning regimen consisted in fludarabine and treosulfan. GVHD prophylaxis consisted in ATG, cyclosporine and a short course of methotrexate for the patient transplanted from MUD and mycophenolate and rapamycin and in the other one. In vivo B cell depletion was also performed with rituximab. Both the patients achieved negative AQ-P4 antibodies and neuroradiological stability without any new lesions documented at MRI follow-up. Also, EDSS dropped from 6 to 3.5 in the first patient and from 8.5 to 7.5 in the second one. The immunological and radiological improvements paralleled clinical improvement with a decreased grade of disability.

Also, our group recently reported the first case of a paediatric patients with NMO treated with an HLA-haploidentical HSCT after ex vivo TCRαβ/CD19 depletion of the graft [8]. Even in this case, the patients achieved clinical, neurological and immunological improvements. Notably, in our case, the disappearance of AQ-P4 antibodies took a long time (6 months after transplantation); however, despite a flare of disease immediately after the transplant, the patient did not experience relapse of the disease. Although the experience is limited, in all cases, alHSCT appeared to be superior in maintaining long-term stabilization of the disease compared to auHSCT.

The reasons for this apparent increased efficiency in the control of the disease could be several. For instance, because of the bidirectional alloreactivity of T cells, in alHSCT, more aggressive myeloablative/immune suppressive regimens are used than auHSCT. This could be more effective in eradicating the patient’s immune system, particularly the autoreactive clones. Moreover, it has been hypothesized that alloreactive T cells of the donor might act similarly to GVHD with a subclinical graft-versus-host anti-autoimmune reaction that could eradicate autoreactive B and T cells. Indeed, both experimental [80] and clinical data [81] support this hypothesis.

**Figure 2:**
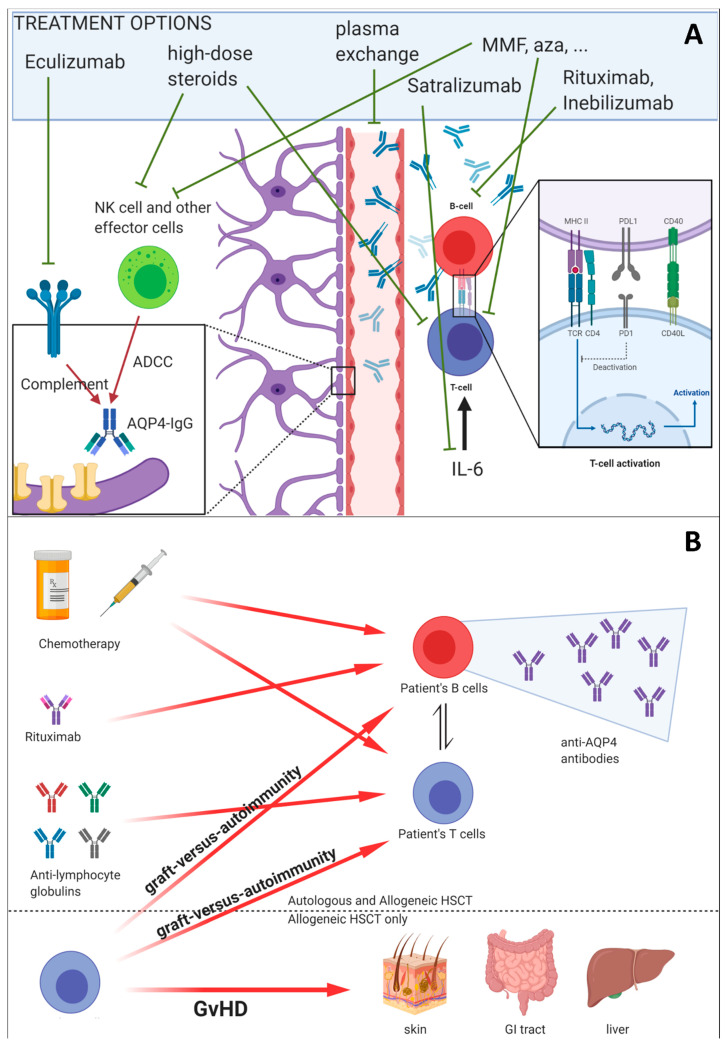
(**A**) Schematic representation of neuromyelitis optica spectrum disorder (NMOSD) pathogenesis: currently available treatments and their targets are reported. ADCC, antibody-dependent cell-mediated cytotoxicity; AQP4-IgG, anti-aquaporin-4 immunoglobulin G; NK, natural killer; MMF, mycophenolate mofetil; aza, azathioprine. (**B**) Allogeneic HSCT rationale and immunological implications.

As already discussed, in the treatment of autoimmune diseases, the lower toxicity of auHSCT has made it the preferred option over alHSCT mainly because of the absence of GVHD and TRM. We have discussed how allogeneic donor T cells may eliminate autoreactive host lymphocytes and therefore may mediate an immunotherapeutic approach (“graft-versus-autoimmunity”) (see also Figure 2). This association was confirmed in a meta-analysis using patients’ data [81]. A paradigmatic case was described by Slavin and colleagues [82]; a patient affected by chronic myelogenous leukaemia and systemic psoriasis with polyarthritis was treated with alHSCT following non-myeloablative conditioning. Initially, both leukaemia and the autoimmune disease were cured. However, later, a recurrence of polyarthritis and psoriasis was observed at the same time as an increase in the proportion of the host DNA (i.e., mixed chimerism). Notably, concomitant reappearance of leukemic minimal residual disease (MRD) was observed. Both molecular MRD positivity and autoimmune manifestations were successfully resolved by the discontinuation of anti-GVHD prophylaxis with Cyclosporine A (CSA) that resulted in the activation of alloreactive T cells leading to GVHD and to Graft-versus-Leukemia (GVL) and graft-versus-autoimmunity effect. This observation corroborates the hypothesis that donor lymphocytes have a role in eliminating self-reactive host T cells. The risk of GVHD has to be carefully evaluated in this context, giving the clinician the hint of a reactive immune system that may also act against the autoimmunity. Indeed, GVHD is a severe and potentially lethal condition, so its manifestations must be promptly recognized and treated. Other complications, including infections, graft rejection, veno-occlusive-disease (VOD) and massive haemorrhage [58], accounting for considerable morbidity and mortality of alHSCT must be carefully weighed against its therapeutic benefits.

## 9. Conclusions

The NMOSD includes clinical pictures of variable severity, many at high risk of recurrence and strongly disabling which might lead to loss of visual or motor functions in a short time. In recent years, much effort has been put into better understanding the pathogenesis of the disease and, in particular, the different immune mechanisms involved in NMOSD besides the production of AQP4- IgG. This has promoted the development of new therapeutic frontiers that can change the course of the disease. Starting from the off-label use of immunosuppressive drugs in NMOSD, more recently, new monoclonal antigens have been tested in specific NMOSD trials. In addition, in the refractory forms of immunosuppressive treatments, HSCT must be addressed and it should be considered as a possible therapeutic option in the most severe form of NMOSD.

## Figures and Tables

**Table 1:** Summary of patients’ characteristics of reported allogeneic hematopoietic stem cells (alHSCT).

Characheristics	Pt1	Pt2	Pt3
Age	30	28	15
Sex	M	F	F
Conditioning	Flu/Threo	Flu/Threo	Flu/Threo/TT
GVHD prophilaxis	ATG/Cyclosporine/Mtx	Mycophenolate/Rapamycin	ATG/αβ depletion
Previous AuHSCT	Yes	Yes (2)	No
EDSS improv	6–3.5	8.5–7.5	6.5–5
AQP4 negativity after HSCT	Yes	Yes	Yes
Donor Type	HLA-id sibling	HLA matched (9/10) MUD	HLA-Haploidentical
Follow-up	3 years	3 years	2 years
Reported in	Greco et al.	Greco et al.	Ceglie et al.

alHSCT, allogeneic HSCT; HSCT, hematopoietic stem cell transplantation; MTX, Methotrexate; Flu, Fludarabine; Threo, Threosulfan; TT, Thiotepa; HLA, Human Leukocyte Antigen; id, identical; MUD, Matched Unrelated Donor; ATG, Antithymocyte Globulin, EDSS, Expanded Disability Status Scale, Pt, Patients.

## Abbreviations

NMO	neuromyelitis optica
NMOSD	neuromyelitis optica spectrum disorders
CNS	central nervous system
HSCT	hematopoietic stem cell transplantation
AQP4	aquaporin-4
CSF	cerebrospinal fluid
MOG-Ab	myelin oligodendrocyte glycoprotein antibody
BBB	the blood–brain barrier
GM-CSF	granulocyte-macrophage colony-stimulating factor
LETM	longitudinally extensive transverse myelitis
MRI	magnetic resonance imaging
AZA	azathioprine
MMF	mycophenolate mofetil
RTX	rituximab
FDA	Food and Drug Administration
EMA	European Medicine Agency
HR	hazard ratio
AuHSCT	autologous HSCT
AlHSCT	allogeneic HSCT
HSCs	hematopoietic stem cells
GVHD	graft versus host disease
TRM	transplant-related mortality
EAE	experimental autoimmune encephalomyelitis
TBI	total body irradiation
Cy	cyclophosphamide
MS	multiple sclerosis
ATG	antithymocyte globulin
EDSS	Expanded Disability Status Scale
OSMS	opticospinal multiple sclerosis
EBMT	European Group for Blood and Marrow Transplantation
AID	autoimmune diseases
HLA	human leukocyte antigen
MFD	matched family donor
MUD	matched unrelated donors
Flu	fludarabine
Threo	threosulfan
VOD	veno-occlusive-disease
